# Multigene phylogeny supports diversification of four-eyed fishes and one-sided livebearers (Cyprinodontiformes: Anablepidae) related to major South American geological events

**DOI:** 10.1371/journal.pone.0199201

**Published:** 2018-06-18

**Authors:** Pedro F. Amorim, Wilson J. E. M. Costa

**Affiliations:** Laboratory of Systematics and Evolution of Teleost Fishes, Institute of Biology, Federal University of Rio de Janeiro, Rio de Janeiro, Rio de Janeiro, Brazil; SOUTHWEST UNIVERSITY, CHINA

## Abstract

The high diversity of Neotropical fishes has been attributed to major South American palaeogeographic events, such as Andean uplift, rise of the Isthmus of Panama and marine transgressions. However, the unavailability of temporal information about evolution and diversification of some fish groups prevents the establishment of robust hypotheses about correlations between species diversification and proposed palaeogeographical events. One example is the Anablepidae, a family of teleost fishes found mostly in coastal habitats of Central and South America, but also in some inner river basins of South America. Historical aspects of the distribution patterns of the Anablepidae were never analysed and no accurate estimation of time of its origin and diversification is presently available. A multi-gene analysis was performed to estimate Anablepidae phylogenetic position, age and biogeography, comprising seven nuclear genes. The suborder Cyprinodontoidei was recovered in three major clades, one comprising all the Old World Cyprinodontoidei and two comprising New World lineages. Anablepidae was recovered as the sister group of the New World Poeciliidae, with the Amazonian genus *Fluviphylax* as their sister group. The ages found for the origin and diversification of Cyprinodontiformes were congruent with the pattern recorded for other vertebrate groups, with an origin anterior to the Cretaceous-Paleogene (K-Pg) transition and diversification during the Paleogene. The age estimated for the split between the Atlantic and Pacific lineages of *Anableps* was congruent with the rise of Panamanian Isthmus. The results suggest Miocene marine transgressions as determinant to the current distribution of *Jenynsia*.

## Introduction

The family Anablepidae is a group of cyprinodontiform fishes comprising three recent genera, *Anableps* Scopoli, 1777, *Jenynsia* Günther, 1866 and *Oxyzygonectes* Fowler, 1916, commonly known as four-eyed fishes, one-sided livebearers, and white-eye, respectively [1]. Two species of *Jenynsia* and all those included in *Anableps* and *Oxyzygonectes* live in coastal salt-water or brackish-water habitats, whereas the remaining 12 species of *Jenynsia* inhabit freshwaters [2, 3, 4, 5, 6].

Regarding the fossil record, a monotypic extinct genus, *Carrionellus* White, 1927 from the Early Miocene of Loja, Ecuador, was first described as a member of the Cyprinodontidae [7], and later transferred to Anablepidae species [2]. However, after a detailed analysis of the type series of *Carrionellus diumortuus* White, 1927, the genus was then realocated in the Cyprinodontidae family [8]. In a recent study two new monotypic genera from Middle Miocene of Northern Argentina were described in the family Anablepidae, *Tucumanableps* Sferco, Herbst, Aguilera & Mirande, 2017, recovered as the sister group of *Anableps*, and *Sachajenynsia* Sferco, Herbst, Aguilera & Mirande, 2017, the sister of all other genera of Anablepidae except *Oxyzygonectes* [9].

Species of *Anableps* exhibit one of the oddest adaptations among Cyprinodontiformes, comprising prominent eyes that rise above their heads, with horizontally divided pupils with an hourglass-like shape. This unique eye morphology allows them to simultaneously see aerial and aquatic environments when swimming with the middle line of the eye at the water surface [10]. These unusual morphological and behavioural traits attracted attention since the first European travellers arrived in America, so records of *Anableps* date from the early 17th century [11], more than 150 years prior to the formal description of the first species of the genus [12]. The complexity of their eye makes these animals important organisms for studies of embryogeny and gene expression (e.g. [13, 14, 15]). Furthermore, due to the presence of a modified anal fin of males in a tubular gonopodium and a placenta-like tissue in females, which together allow internal fertilization and viviparity [16, 17], *Anableps* and *Jenynsia* are also models of studies involving reproduction and ontogeny (e.g. [18, 19, 20]).

**Fig 1:**
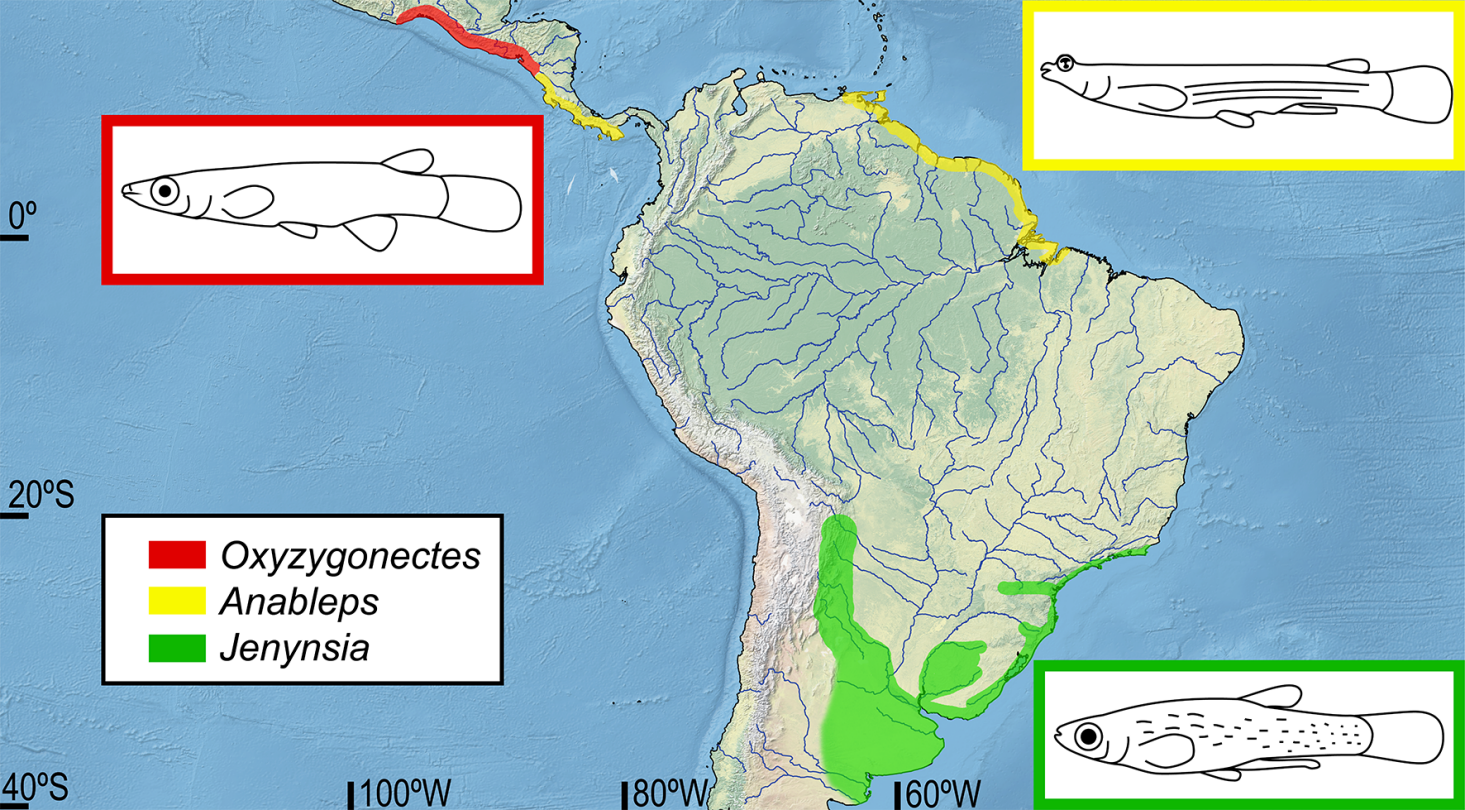
Geographical distribution of the genera of Anablepidae. *Oxyzygonectes* in red, *Anableps* in yellow and *Jenynsia* in green.

The geographical distribution of Anablepidae is restricted to Neotropical areas (Fig 1): *Oxyzygonectes* is a monotypic genus found in the Pacific coast of Central America; *Anableps* comprises three species inhabiting both the Pacific coast of Central America and the Atlantic coast of northern South America; and *Jenynsia*, the most species diverse genus of Anablepidae, comprising 14 species inhabiting the Paraná-Paraguay river system and coastal drainages of south-eastern South America [2, 4, 21]. However, biogeographical historical events responsible for the present distribution of the Anablepidae were never deeply explored [2]. Previous authors discussed the distribution of *Anableps* which could be related to the rise of the Panama Isthmus or ancient events, but no conclusion was proposed [1, 2, 5]. *Jenynsia* presents a huge disjunct distribution when compared to other anablepid genera (Fig 1), but there is no available explanation for this distribution pattern based on analytical tools for biogeographical inference. Here we first provide a time-calibrated multi-gene phylogeny of the Anablepidae, discussing two possible past historical scenarios that have shaped the present distribution of the main Anablepidae components: (1) invasion of the most recent common ancestor into the Amazon basin through Miocene marine transgressions followed by dispersion to south through ancient connections between the Amazon and Paraná river basins or (2) a dispersion along the eastern South America coast with an invasion to the freshwater habitats in the mouth of the La Plata river, during Miocene, through marine transgression forming the Paranean Sea.

Age estimates for the Anablepidae and other Neotropical cyprinodontoid groups are still uncertain. Previous studies proposed the origin of the clade comprising Anablepidae and Poeciliidae (sensu [1]) dating back to the Lower Cretaceous (130–110 Ma), before separation between Africa and South America [22, 23, 24], but a recent time-calibrated analysis [25] estimated the origin of Poecilinae and Anablepidae clade between the Upper Cretaceous and the Early Palaeocene (62–67 Ma). In the current study, we analysed the largest dataset of genetic information for Anablepidae to test its phylogenetic position among the Cyprinodontoidei and to estimate time of origin and diversification of the family and their genera using three calibrating points based on well-known cyprinodontoid fossils.

## Materials and methods

### Taxonomic sampling, DNA extraction, PCR, and sequencing

A total of 82 terminal taxa were used, including one species of Beloniformes, the sister group to Cyprinodontiformes (e.g. [26, 27]), in which the analyses were rooted, and 81 species of Cyprinodontiformes, among which are represented all species of *Anableps* and *Oxyzygonectes*, and eight of the 14 species of *Jenynsia*. Molecular data were obtained from specimens euthanized with buffered solution of ethyl-3-amino-benzoat-methansulfonat (MS-222) at a concentration of 250 mg/l, for a period of 10 minutes or more, until completely ceasing opercular movements, following the methods for euthanasia approved by CEUA-CCS-UFRJ (Ethics Committee for Animal Use of Federal University of Rio de Janeiro; permit number: 01200.001568/2013-87). These individuals were fixed and preserved in absolute ethanol. The dataset was complemented with sequences obtained from GenBank, a complete list of taxa used in the analysis is presented in S1 Table. Seven nuclear genes were sampled: ectodermal-neural cortex (ENC1), glycosyltransferase (GLYT), cardiac muscle myosin heavy chain 6 alpha (MYH6), recombination activating gene 1 (RAG1), rhodopsin (RHO), SH3 and PX domain-containing 3-like protein (SH3PX3) and tyrosine kinase exons 8–10 and introns 8–9 (X-SRC). The introns of X-SRC were excluded due to the high degree of variability found, which produce highly ambiguous alignments.

Total genomic DNA was extracted from muscle tissue of the right side of the caudal peduncle using the DNeasy Blood & Tissue Kit (Qiagen), according to the manufacturer instructions. To amplify the fragments of the DNA, the primers provided in the S2 Table were used. Polymerase chain reactions (PCR) were performed in 30μl reaction mixtures containing 5x Green GoTaq Reaction Buffer (Promega), 3.2 mM MgCl_2_, 1 μM of each primer, 75 ng of total genomic DNA, 0.2 mM of each dNTP and 1U of Taq polymerase. The thermocycling profile was: (1) 1 cycle of 4 minutes at 94°C; (2) 35 cycles of 1 minute at 92°C, 1 minute at 50–63°C and 1 minute at 72°C; and (3) 1 cycle of 4 minutes at 72°C. In all PCR reactions, negative controls without DNA were used to check contaminations. Amplified PCR products were purified using the Wizard SV Gel and PCR Clean-Up System (Promega). Sequencing reactions were made by Macrogen Inc. (South Korea) using the BigDye Terminator Cycle Sequencing Mix (Applied Biosystems). Cycle sequencing reactions were performed in 10 μl reaction volumes containing 1 μl BigDye 2.5, 1.55 μl 5x sequencing buffer (Applied Biosystems), 2 μl of the amplified products (10–40ng), and 2 μl primer. The thermocycling profile was: (1) 35 cycles of 10 seconds at 96°C, 5 seconds at 54°C and 4 minutes at 60°C. The sequencing reactions were purified and denatured and the samples were run on an ABI 3130 Genetic Analyzer. Sequences were edited using MEGA 6 [28] and aligned using ClustalW [29]. The DNA sequences were translated into amino acids residues to test for the absence of premature stop codons or indels using the program MEGA 6.0.

### Phylogenetic analysis

The dataset was analysed in the program PartitionFinder [30] to determine the best partitioning scheme and nucleotide substitution models. The optimal partition strategy is shown in S3 Table. The phylogenetic analyses were performed in the programs Garli 2.0 [31], for Maximum Likelyhood (ML) and MrBayes 3.2.6 [32], for Bayesian inference (BI). The support values of the ML analysis were calculated by 1000 bootstrap replications [33]. For BI analysis, four independent Markov Chain Monte Carlo (MCMC) were performed with 30 million generations each, sampling one of every 1,000 trees. The support values of the BI analysis are given by posterior probability. The quality of the MCMC chains was evaluated in Tracer 1.6 [34], and a 25% burn-in was removed.

### Divergence-time estimation

**Fig 2:**
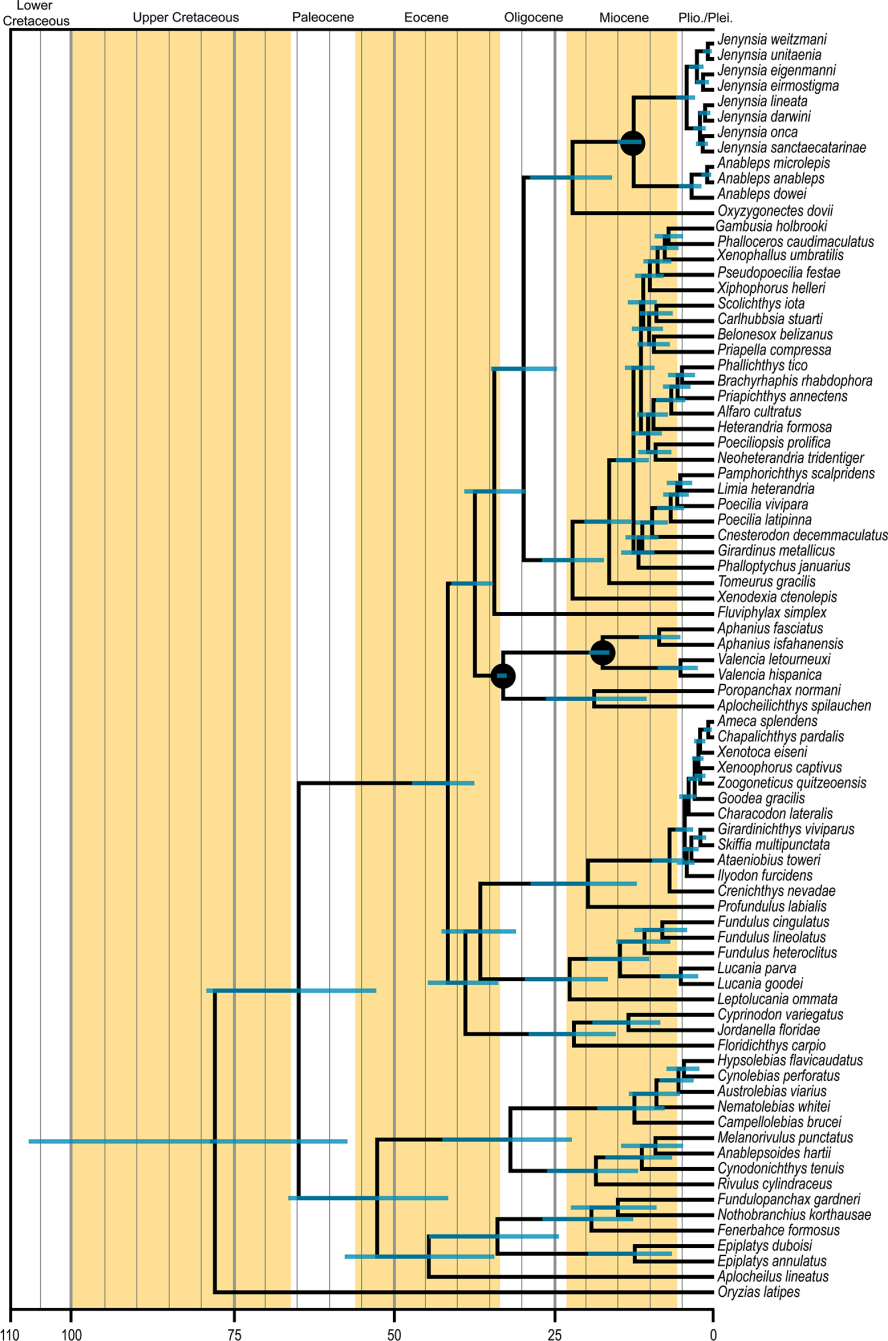
Time-calibrated analysis performed in Beast 1.8.3. Black circles indicate the position of the calibrations used. Bars represent the 95% highest posterior credibility intervals of divergence times.

The divergence date analyses were performed in Beast v.1.8 [35, 36], using the concatenated dataset and the same partitions as described above, and a normal uncorrelated relaxed clock model, which emphasizes the minimum age and has been considered appropriate for fossil calibration points [37]. Bayesian inference was performed with 30 million generations, a sampling frequency of 1000. The value of parameters of the analysis, convergence of the MCMC chains, sample size and the stationary phase of the chains were evaluated using Tracer v. 1.6 [34]. A Yule speciation process for the tree prior [38] was used, establishing three calibration points. The first one was placed at the stem comprising the genera *Aphanius* and *Valencia*. It corresponds to the origin of the crown European cyprinodontoid clade [39], which was estimated to have occurred at least 33 Ma on the basis of the oldest identifiable clade member, the fossil *Prolebias stenoura* Sauvage, 1874 from the Lower Stampien (Lower Oligocene) of Puy-de-Dôme, France [40] (prior setting: lognormal distribution, mean = 33 and standard deviation = 0.5). The second point was the node where *Aphanius* and *Valencia* diverge, corresponding to the most ancient record with recognizable synapomorphies of *Aphanius*, the fossil *Aphanius chebianus* (Obrhelová, 1985) of the Ottnangian (Lower Miocene) of the Cheb basin, Czech Republic ([41]; WJEMC, pers. observ.), with 17 Ma (prior setting: lognormal distribution, mean = 17 and standard deviation = 0.5). The third point was placed in the divergence between *Anableps* and *Jenynsia*, this position corresponds to the *Tucumanableps cionei* Sferco, Herbst, Aguilera & Mirande, 2017. This species is recognised as the sister goup of *Anabelps* from the Meddle Miocene of the Río Sali formation, Argentina [9], with 12 Ma (prior settings: lognormal distribution, mean = 12 and standard deviation = 0.5). The position of each calibration points is also shown in Fig 2.

### Reconstructing ancestral habitat

An analysis of ancestral states was used to infer possible past habitat scenarios without *a priori* assumptions about states relationships [42]. Reconstruction of ancestral states were conducted in the program RASP 3.02 [43] using the analytical approach Bayesian Binary MCMC (BBM) [44]. The analysis was directed to historical pattern of habitats, based on: (A) freshwater and (B) brackish water.

## Results

### Phylogenetic relationships

**Fig 3:**
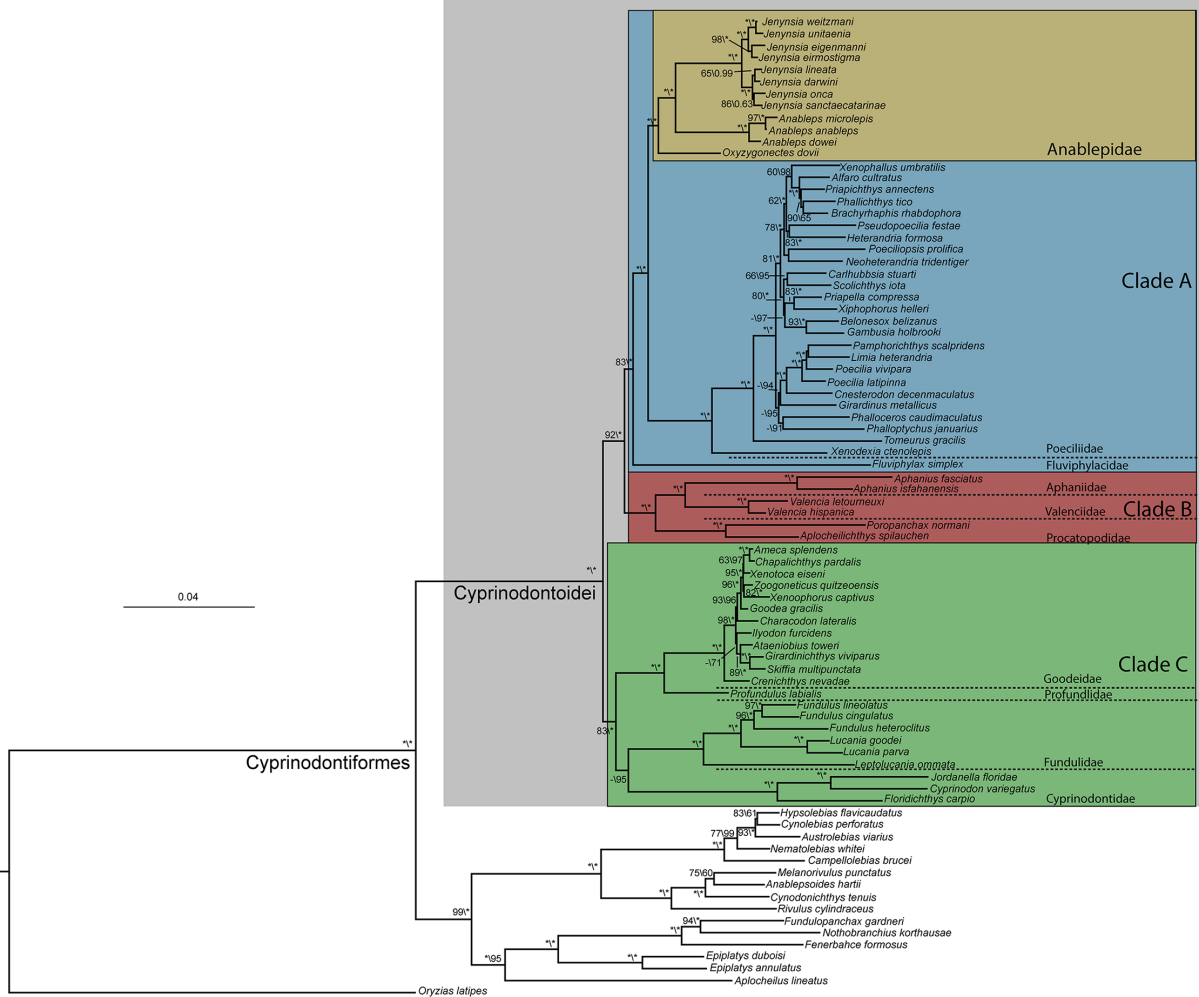
Maximum likelihood (ML) tree. ML tree with the same topology of the Bayesian Inference (BI) tree. Values on the nodes are relative to the Bootstrap of ML and posterior probability of BI analyses respectively. Asterisks mean maximum values and dashes mean values under 60%.

The analyses of BI and ML (Fig 3) presented identical topologies and high supports for most nodes. Both suborders of Cyprinodontiformes, Cyprinodontoidei, and Aplocheiloidei, were recovered with high support values. Three main clades were found in Cyprinodontoidei, here referred as clades A, B and C, clades A and C comprising New World taxa and clade B restricted to Old World taxa (Fig 3). Both analyses did not recover Poeciliidae (sensu [1]) as monophyletic, with their taxa appearing in three unrelated lineages: one, including African procatopodines as part of the Old-World clade B, and two, comprising the subfamily Poeciliinae and the Amazonian genus *Fluviphylax* as unrelated parts of the New World clade A (Fig 3). The analyses highly supported Anablepidae as sister to the internal fertilizing family Poeciliidae (sensu [45]), as well as a clade containing Anablepidae, Poeciliidae, and *Fluviphylax* (clade A). In Anablepidae the genus *Oxyzygonectes* was recovered as the sister group of *Anableps* and *Jenynsia*, and both subgenera of *Jenynsia* were also corroborated [2].

### Time-calibrated analysis

The Beast 1.8.3 time-calibrated analysis is shown in Fig 2. Divergence times and their estimated 95% highest posterior density (HPD) intervals place the origin of Cyprinodontiformes in the Upper Cretaceous (77.6 Ma). Subsequent diversification of Cyprinodontoidei in the clades A, B and C occurred in Eocene (between 41.4–37.2 Ma). The currently recognised families originated between Eocene and Miocene (38.1–19.6 Ma).

### Reconstructing ancestral habitat

**Fig 4:**
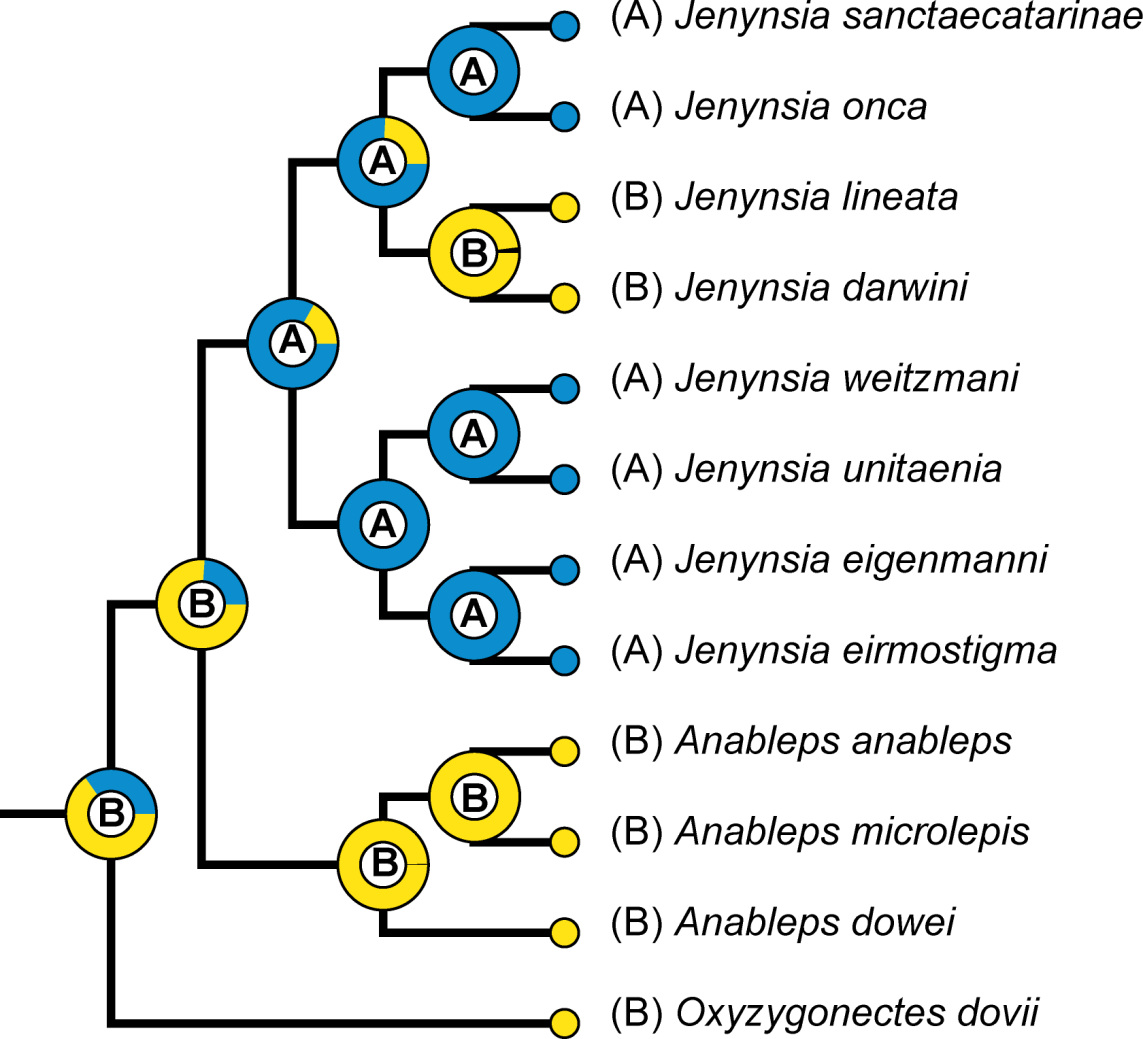
Analysis of evolution of ancestral habitat. BBM analysis based on the Bayesian Inference (BI) tree. Blue indicates freshwater (A), and yellow indicates brackish water (B). Only Anablepidae is shown.

The reconstruction of ancestral habitats recovered the ancestor of Anablepidae as a brackish water species (Fig 4). In the genus *Jenynsia*, the BBM analysis recovered the most recent common ancestor as a freshwater species, a condition kept in derived lineages, except in the clade comprising *J*. *lineata* and *J*. *darwini*, wherein the analysis recovered the ancestor inhabiting brackish waters.

## Discussion

### Phylogenetic relationships of Anablepidae

The present study is congruent with most recent analyses of Cyprinodontiformes (e.g. [25, 39, 45]), where Poeciliidae (sensu [1],) is not corroborated. Similarly, the phylogenetic analyses recovered three unrelated clades, presently receiving family status, Fluviphylacidae, Poeciliidae and Procatopodidae [45] (Fig 3). Once again, the African clade Procatopodidae was recovered as the sister group of a clade comprising Valenciidae and Aphaniidae [39, 45, 46], composing the Clade B, and the New World lineages Poeciliidae and Fluviphylacidae, recovered in the Clade A along with the Anablepidae (Fig 3). The proposed sister group relationships between Anablepidae and Poeciliinae have been reported since the first molecular phylogenetic analyses of Cyprinodontiformes [25, 47, 48], but the position of *Fluviphylax* as the sister group of this clade was only suggested in recent studies [25, 45].

In addition, this study supports relationships among genera of Anablepidae as proposed in analyses exclusively based on morphological characters [1, 2], with *Oxyzygonectes* as the sister group of the clade containing *Jenynsia* and *Anableps*.

### Timing of origin and diversification of the Anablepidae

In contrast to previous phylogenetic studies where the distribution of the Cyprinodontoidei in both the New and Old World was assumed to be result of continental drift between Africa and South America in the Cretaceous [22, 24], the current time-calibrated analysis (see below) indicated that the whole order Cyprinodontiformes had its origin in the Upper Cretaceous (77.6 Ma, 95% HPD 106.6–57.0 Ma) and the diversification of the Cyprinodontoidei occurred in the Eocene (41.4 Ma, 95% HPD 47.0–37.3 Ma), therefore much more recently than the complete separation of Africa and South America (about 100 Ma). The origin of the Cyprinodontiformes clade just before the Cretaceous-Paleogene transition (K–Pg), followed by a great diversification during the Paleogene, is a pattern also found in other animal groups, such as placental mammals, crocodyliforms, birds and some other groups of teleost fishes (e.g. [49, 50, 51, 52, 53]). The K–Pg event has been associated with a mass extinction event that affected great part of Earth's biodiversity, as well as considered as an important event for the subsequent diversification of vertebrates [49, 54, 55, 56]. The results of the present analyses and the biogeographic information previously published for Poeciliidae [25] indicates that the origin of Anablepidae, and the clade composed by both families, have probably occurred in South America. The origin of the Anablepidae is here estimated to have occurred during the Oligocene (29.6 Ma, 95% HPD 34.7–24.5 Ma), followed by a split between the oviparous brackish and marine genus *Oxyzygonectes* and the clade of viviparous anablepids comprising the brackish and marine genus *Anableps* and the predominantly freshwater genus *Jenynsia* (27.9 Ma, 95% HPD 15.0–12.3 Ma), which diverged from each other in the Miocene (12.5 Ma, 95% HPD 28.7–16.2 Ma). The last time estimate is thus temporally congruent with the Miocene marine transgressions in the Amazon basin [57, 58, 59]. The transition of South American marine fish lineages to freshwater environments have been associated with Miocene marine transgressions, followed by species diversification in freshwater habitats, as the Pebas Mega-wetland system in the Amazon river basin and the Paranean Sea in the Paraná river basin [57, 58]. Those systems were complex ecosystems with different kinds of habitats, promoting diversification of vertebrate and invertebrate lineages and adaptation to freshwater environments [60, 61, 62]. More recently an analysis concluded that marine to freshwater transition in South American drums and pufferfishes was temporally coincident with Miocene marine transgressions in the Amazon river basin [63]. Considering the result of the BBM analysis and that species of *Anableps* and *Oxyzygonectes* are restricted to coastal marine habitats of Central America and northern South America (Fig 4), we postulate that *Jenynsia* is a marine-derived lineage, the origin of the genus is associated with a transition of the ancestor from marine to freshwater environments favoured by Miocene marine transgressions.

The present analysis found that the current geographical range of species of *Jenynsia*, (Fig 1) [2, 21], may be a consequence of an invasion to the freshwater environments and subsequent dispersal through the Pebas Mega-wetland system, combined with an ancient connection between the Amazon and Paraná river basins [59, 64, 65]. Despite some divergences [61, 62, 63, 64, 65, 66], most authors agree that this connection lasted until the Late Miocene (11–10 Ma) [59, 64, 65]. According to the time-calibrated analysis (Fig 2), species diversification of *Jenynsia* occurred in the Early Pliocene (4.3 Ma, 95% HPD 5.9–3.0 Ma), after separation of the Amazon and Paraná river basins. The most recent ancestor of *Jenynsia* must have dispersed along this ancient connection and subsequently invading the Paranean Sea, then reaching the current distribution of the genus, which is partially coincident with the geographical range of this marine transgression in the Paraná river basin from Middle to Late Miocene [21, 67]. The diversification of *Jenynsia* in Early Pliocene (4.6 Ma, 95% HPD 6.5–3.1 Ma) is congruent with the regression of the Paranean Sea [59, 68], so that the current range of the species of *Jenynsia* would be the result of the decrease of this marine transgression, as previously proposed for some of the species of genus [67]. The present distribution pattern of *Jenynsia* is also similar to that found for other aquatic organisms which the distribution is attributed to the Paranean Sea, including the freshwater Crustacean families Palaemonidae, Sergestidae and Trichodactylidae [69]. Both known fossil genera of Anablepiadae were found in an area previously occupied by the Paranean Sea [9]. The presence of *Tucumanableps*, the sister group of *Anableps*, and *Sachajenynsia*, the sister group of all genera of Anablepidae except *Oxyzygonectes*, in this area also corroborates the ancient connections between Amazon and Paraná river basins. This also indicates that the ancestors of *Jenynsia* and other Anablepidea fossil species inhabited Pebas Mega-wetland and Paranean Sea.The time-calibrated analysis inferred the divergence between *A*. *dowei*, endemic to the Pacific coast of Central America, and the clade comprising *A*. *anableps* and *A*. *microlepis*, two species endemic to northern South American coast, occurred in the Early Pliocene (3.5 Ma, 95% HPD 5.5–2.0 Ma). This age is coincident with the final stages of the rise of the Panamanian Isthmus [70], as previously suggested [2, 5]. However, due to the complex geological history of Central America and the absence of time information, previously authors argued that this disjunctive distribution pattern could also be caused by older events. The present time-calibrated analysis is the first to highly supports that disjunction as a result of the rise of the Panamanian Isthmus.

The only known fossil record of a recent anablepid genus consists of a premaxilla belonging to an unidentified species of *Jenynsia* from the Late Pleistocene (230–125 Kyr) of Centinela del Mar, Argentina [71]. The only extant anablepid species found in this area is *J*. *lineata*, for which an age of 1.4 Ma (95% HPD 2.4–0.6 Ma; Pleistocene) is herein estimated. Considering the premaxilla morphology, the site localization, and the fossil age, we conclude that this fossil probably represents a specimen of *J*. *lineata* or an extinct closely related species.

## Supporting information

S1 Table List of analysed species with respective GenBank accession numbers for each analysed gene. Bold accession numbers are sequences first used in this study.
(DOC)

S2 Table List of primers for each gene. (DOC)

S3 Table Best evolutive model for each partition found by PartitionFinder. (DOC)
