# Supplementary material for: Multigene phylogeny supports diversification of four-eyed fishes and one-sided livebearers (Cyprinodontiformes: Anablepidae) related to major South American geological events
Source: PLoS One. 2018 Jun 18;13(6):e0199201. doi: 10.1371/journal.pone.0199201 (PMC6005514; doi:10.1371/journal.pone.0199201)
Supplement: S1 Table — Bold accession numbers are sequences first used in this study. (DOC) [file pone.0199201.s001.doc]

S1 Table. List of analysed species with respective GenBank accession numbers for each analysed gene. Bold accession numbers are sequences first used in this study.

| Species | Voucher | **ENC1** | **GLYT** | **MYH6** | **RAG1** | **RHO** | **SH3PX3** | **X-SRC** |
| --- | --- | --- | --- | --- | --- | --- | --- | --- |
| *Oryzias latipes* | - | EF032979 | EF032992 | EF032927 | EF095641 | XM 004081490 | EF033005 | - |
| *Anablepsoides hartii* | - | KJ696966 | KJ697076 | KJ697186 | - | KJ697449 | KJ697559 | KJ697665 |
| ***Nematolebias whitei*** | **UFRJ 6844** | KC701991 | KJ844639 | **KY927083** | KJ844686 | KC702057 | **KY031609** | U02348 |
| *Austrolebias viarius* | - | KY031578 | KY093062 | - | JX566801 | KY076590 | KY031607 | - |
| *Campellolebias brucei* | - | KY031589 | KT590083 | - | - | KY076601 | KY031613 | - |
| *Cynodonichthys tenuis* | - | KC702024 | - | - | KJ844673 | KC702089 | KC702154 | - |
| *Cynolebias perforatus* | - | KY031571 | KY093052 | - | - | KY076581 | KY031599 | - |
| *Rivulus cylindraceu* | - | KC702013 | KJ844657 | - | KJ844703 | KC702077 | KC702143 | - |
| *Hypsolebias flavicaudatus* | - | KY031569 | KY093046 | - | - | KY076574 | KY031597 | - |
| *Melanorivulus punctatus* | - | KC702021 | KY093073 | - | - | KC702087 | KY031615 | - |
| *Nothobranchius korthausae* | - | HAEB01011967 | KJ844641 | KJ179410 | KJ844688 | HAEB01019998 | KJ179469 | - |
| *Epiplatys duboisi* | - | KJ696872 | KJ696981 | KJ697091 | KJ697270 | KJ697355 | KJ697464 | KJ697574 |
| *Epiplatys annulatus* | - | KJ696886 | KJ696996 | KJ697106 | KJ697279 | KJ697369 | KJ697479 | - |
| *Fenerbahce formosus* | - | KJ696867 | KJ696976 | KJ697086 | KJ697266 | KJ697350 | KJ697459 | - |
| *Fundulopanchax gardineri* | - | KJ696889 | KJ696999 | KJ697109 | KJ697281 | KJ697372 | KJ697482 | KJ697588 |
| *Aplocheilus lineatus* | - | KC701974 | KJ844619 | KJ697095 | KJ697274 | KC702042 | KJ697468 | - |
| *Profundulus labialis* | - | KJ696964 | KJ697074 | KJ697184 | - | KJ697447 | KJ697557 | - |
| *Crenichthys nevadae* | - | KJ696882 | KJ696992 | KJ697102 | KJ697278 | KJ697365 | KJ697475 | KJ697583 |
| ***Characodon lateralis*** | **UFRJ 10884** | **KY927057** | **KY927070** | **KY927084** | **KY927098** | **KY927112** | **KY986648** | **KY927126** |
| ***Ameca splendens*** | **UFRJ 10885** | **KY927058** | **KY927072** | **KY927085** | **KY927099** | **KY927113** | **KY986649** | **KY927127** |
| *Goodea gracilis* | - | KJ696896 | KJ697006 | KJ697116 | KJ697287 | KJ697379 | KJ697489 | KJ697595 |
| *Zoogoneticus quitzeoensis* | - | KJ696975 | KJ697085 | KJ697195 | KJ697349 | KJ697458 | KJ697568 | U02366 |
| *Xenotoca eiseni* | - | KJ696974 | KJ697084 | KJ697194 | KJ697348 | KJ697457 | KJ697567 | U02360 |
| *Xenoophorus captivus* | - | KJ696972 | KJ697082 | KJ697192 | KJ697346 | KJ697455 | KJ697565 | KJ697671 |
| *Skiffia multipunctata* | - | KJ696968 | KJ697078 | KJ697188 | KJ697345 | KJ697451 | KJ697561 | KJ697667 |
| *Ilyodon furcidens* | - | KJ696898 | KJ697008 | KJ697118 | KJ697288 | KJ697381 | KJ697491 | KJ697597 |
| *Girardinichthys viviparus* | - | KJ696894 | KJ697004 | KJ697114 | KJ697286 | KJ697377 | KJ697487 | KJ697593 |
| *Chapalichthys pardalis* | - | KJ696880 | KJ696990 | KJ697100 | KJ697276 | KJ697363 | KJ697473 | KJ697581 |
| *Ataeniobius toweri* | - | KJ696876 | KJ696986 | KJ697096 | KJ697275 | KJ697359 | KJ697469 | KJ697577 |
| *Leptolucania ommata* | **UFRJ 10883** | **KY927059** | **KY927071** | **KY927086** | **KY927100** | **KY927114** | - | **KY927128** |
| *Lucania goodei* | - | KJ696915 | KJ697025 | KJ697135 | KJ697304 | KJ697398 | KJ697508 | KJ697614 |
| *Lucania parva* | - | KJ696916 | KJ697026 | KJ697136 | GQ119934 | KJ697399 | KJ697509 | KJ697615 |
| *Funulus heteroclitus* | - | EF032978 | EF032991 | EF032926 | GQ119889 | KJ697374 | EF033004 | U02351 |
| *Fundulus lineolatus* | - | KJ696891 | KJ697001 | KJ697111 | KJ697283 | KJ697374 | KJ697484 | KJ697590 |
| *Fundulus cingulatus* | - | KJ696890 | KJ697000 | KJ697110 | KJ697282 | KJ697373 | KJ697483 | KJ697589 |
| *Floridichthys carpio* | - | KJ696887 | KJ696997 | KJ697107 | - | KJ697370 | KJ697480 | KJ697586 |
| *Jordanella floridae* | - | KJ696901 | KJ697011 | KJ697121 | KF141266 | KJ697384 | KJ697494 | KJ697600 |
| *Cyprinodon variegatus* | - | KJ696885 | KJ696995 | KJ697105 | KF141215 | KJ697368 | KJ697478 | KJ697585 |
| ***Aphanius fasciatus*** | **UFRJ 8076** | **KY927060** | **KY927073** | **KY927087** | **KY927101** | **KY927115** | - | - |
| ***Aphanius isfahanensis*** | **UFRJ 8079** | **KY927061** | **KY927074** | **KY927088** | **KY927102** | **KY927116** | - | **KY927129** |
| *Valencia hispanica* | - | KJ696970 | KJ697080 | KJ697190 | - | KJ697453 | KJ697563 | KJ697669 |
| ***Valencia letourneuxi*** | **UFRJ 8108** | **KY927062** | **KY927075** | **KY927089** | **KY927103** | **KY927117** | **KY986650** | **KY927130** |
| *Aplocheilichthys spilouchen* | - | KJ696874 | KJ696984 | KJ697094 | KJ697273 | KJ697357 | KJ697467 | U02344 |
| *Poropanchax normani* | - | KJ696873 | KJ696983 | KJ697093 | KJ697272 | KJ697356 | KJ697466 | KJ697575 |
| Fluviphylax simplex | - | KJ696888 | KJ696998 | KJ697108 | KJ697280 | KJ697371 | KJ697481 | KJ697587 |
| Xiphophorus helleri | - | KJ525879 | KJ525859 | KJ525839 | EF017445 | KJ525799 | KJ525779 | KJ525899 |
| Belonesox belisanus | - | KJ696877 | KJ696987 | KJ697097 | EF017416 | KJ697360 | KJ697470 | KJ697578 |
| Phaloceros cadimaculatus | - | KJ696926 | KJ697036 | KJ697146 | EF017426 | KJ697409 | KJ697519 | KJ697625 |
| Poecilia vivipara | - | HQ857473 | HQ857467 | HQ857461 | HQ857449 | HQ857443 | HQ857425 | HQ857437 |
| Cnesteredon decenmaculatus | - | GU179168 | GU179197 | GU179243 | EF017427 | GU179271 | GU179214 | GU179152 |
| Alfaro cultratus | - | KJ696868 | KJ696977 | KJ697087 | -- | KJ697351 | KJ697460 | KJ697570 |
| Phalloptychus januarius | - | KJ696927 | KJ697037 | KJ697147 | EF017428 | KJ697410 | KJ697520 | KJ697626 |
| Poeciliopsis prolifica | - | KJ696954 | KJ697064 | KJ697174 | KJ697336 | KJ697437 | KJ697547 | KJ697653 |
| Tomeurus gracilis | - | KJ696969 | KJ697079 | KJ697189 | EF017455 | KJ697452 | KJ697562 | U02359 |
| Xenodexia ctenoleps | - | KJ696971 | KJ697081 | KJ697191 | EF017454 | KJ697454 | KJ697564 | KJ697670 |
| Gambusia holbrooki | - | KJ696892 | KJ697002 | KJ697112 | KJ697284 | KJ697375 | KJ697485 | - |
| Limia heterandria | - | HQ857468 | HQ857462 | HQ857456 | HQ857444 | HQ857438 | HQ857420 | HQ857432 |
| Xenophallus umbratilis | - | KJ696973 | KJ697083 | KJ697193 | KJ697347 | KJ697456 | KJ697566 | KJ697672 |
| Scolichthys iota | - | KJ696967 | KJ697077 | KJ697187 | EF017438 | KJ697450 | KJ697560 | KJ697666 |
| Pseudopoecilia festae | - | KJ696965 | KJ697075 | KJ697185 | EF017440 | KJ697448 | KJ697558 | KJ697664 |
| Poecilia latipinna | - | KJ696932 | KJ697042 | KJ697152 | KJ697314 | KJ697415 | KJ697525 | KJ697631 |
| Brachyrhaphis rhabdophora | - | KJ696878 | KJ696988 | KJ697098 | EF017419 | KJ697361 | KJ697471 | KJ697579 |
| Phallichthys tico | - | KJ696925 | KJ697035 | KJ697145 | EF017409 | KJ697408 | KJ697518 | KJ697624 |
| Priapella compressa | - | KJ525891 | KJ525871 | KJ525851 | KJ525831 | KJ525811 | KJ525791 | KJ525911 |
| Carlhubbsia stuarti | - | KJ696879 | KJ696989 | KJ697099 | EF017430 | KJ697362 | KJ697472 | KJ697580 |
| Girardinus metallicus | - | KJ696895 | KJ697005 | KJ697115 | EF017441 | KJ697378 | KJ697488 | KJ697594 |
| Priapichthys annectens | - | KJ696961 | KJ697071 | KJ697181 | EF017439 | KJ697444 | KJ697554 | KJ697660 |
| Pamphorichthys scalpridens | - | HQ857471 | HQ857465 | HQ857459 | HQ857447 | HQ857441 | HQ857423 | HQ857435 |
| Neoheterandria tridentiger | - | KJ696920 | KJ697030 | KJ697140 | EF017423 | KJ697403 | KJ697513 | KJ697619 |
| Heterandria formosa | - | KJ696897 | KJ697007 | KJ697117 | EF017422 | KJ697380 | KJ697490 | KJ697596 |
| Oxyzygonectes dovii | - | KJ696922 | KJ697032 | KJ697142 | KJ697307 | KJ697405 | KJ697515 | KJ697621 |
| Anableps dowei | - | KJ696871 | KJ696980 | KJ697090 | KJ697269 | KJ697354 | KJ697463 | KJ697573 |
| Anableps anableps | - | KJ696870 | KJ696979 | KJ697089 | EF017405 | KJ697353 | KJ697462 | KJ697572 |
| **Anableps microleps** | **UFRJ 8004** | **KY927063** | **KY927076** | **KY927090** | **KY927104** | **KY927118** | **KY986651** | **KY927131** |
| **Jenynsia eirmostigma** | **UFRJ 10561** | **KY927064** | **KY927077** | **KY927091** | **KY927105** | **KY927119** | **KY986652** | **KY927132** |
| **Jenynsia eignemanni** | **UFRJ 10832** | - | - | **KY927092** | **KY927106** | **KY927120** | **KY986653** | **KY927133** |
| **Jenynsia unitaenia** | **UFRJ 10190** | **KY927065** | **KY927078** | **KY927093** | **KY927107** | **KY927121** | **KY031617** | **KY927134** |
| **Jenynsia weitzmani** | **UFRJ 10643** | **KY927066** | **KY927079** | **KY927094** | **KY927108** | **KY927122** | **KY986654** | **KY927135** |
| **Jenynsia sp** | **UFRJ 10034** | **KY927067** | **KY927080** | **KY927095** | **KY927109** | **KY927123** | **KY986655** | **KY927136** |
| **Jenynsia sactaecatarinae** | **UFRJ 10564** | **KY927068** | **KY927081** | **KY927096** | **KY927110** | **KY927124** | **KY986656** | **KY927137** |
| Jenynsia lineata | - | KJ696900 | KJ697010 | KJ697120 | KJ697290 | KJ697383 | KJ697493 | KJ697599 |
| **Jenynsia onca** | **ZVCP 13633** | **KY927069** | **KY927082** | **KY927097** | **KY927111** | **KY927125** | **KY986657** | **KY927138** |
