# Supplementary material for: Multigene phylogeny supports diversification of four-eyed fishes and one-sided livebearers (Cyprinodontiformes: Anablepidae) related to major South American geological events
Source: PLoS One. 2018 Jun 18;13(6):e0199201. doi: 10.1371/journal.pone.0199201 (PMC6005514; doi:10.1371/journal.pone.0199201)
Supplement: S2 Table — (DOC) [file pone.0199201.s002.doc]

S2 Table. List of primers for each gene.

| **Gene** | **Primer** | **Sequence** | **Reference** |
| --- | --- | --- | --- |
| ENC1 | ENC1_F85 | GACATGCTGGAGTTTCAGGA | [1] |
|  | ENC1_F88 | ATGCTGGAGTTTCAGGACAT | [1] |
|  | ENC1_R975 | AGCMACTGGGTCAAACTGCTC | [1] |
|  | ENC1_R982 | ACTTGTTRGCMACTGGGTCAAA | [1] |
| GLYT | GLYT_F577 | ACATGGTACCAGTATGGCTTTGT | [1] |
|  | GLYT_F559 | GGACTGTCMAAGATGACCACMT | [1] |
|  | GLYT_R1562 | CCCAAGAGGTTCTTGTTRAAGAT | [1] |
| MYH6 | myh6_F507 | GGAGAATCARTCKGTGCTCATCA | [1] |
|  | myh6_R1325 | ATTCTCACCACCATCCAGTTGAA | [1] |
| RAG1 | RAG1F1 | CTGAGCTGCAGTCAGTACCATAAGATG | [2] |
|  | RAG1R2 | TGAGTCCTTGTGAGCTTCCATRAAYTT | [2] |
|  | H3405 | GCNGAGACTCCTTTGACTCTGTC | [3] |
| RHO | Rh545 | GCAAGCCCATCAGCAACTTCCG | [4] |
|  | Rh1039R | TGCTTGTTCATGCAGATGTAGA | [4] |
| SH3PX3 | SH3PX3_F532 | GACGTTCCCATGATGGCWAAAAT | [1] |
|  | SH3PX3_R1303 | CAAACAKCTCYCCGATGTTCTC | [1] |
| X-SRC | X-SRCC | CTCAATCAGGCGAGCCAACCAAAATC | [5] |
|  | X-SRCD | ACGGCACCACACAGGTGGCGATCAA | [5] |

References

1. Li C, Ortí G, Zhang G, Lu G. A practical approach to phylogenomics: The phylogeny of ray-finned fish (Actinopterygii) as a case study. BMC Evol Biol. 2007; 7: 33–44.
2. López JA, Chen WJ, Ortí G. Esociform phylogeny. Copeia. 2004; 2004: 449–464.
3. Hrbek T, Seckinger J, Meyer A. A phylogenetic and biogeographic perspective on the evolution of poeciliid fishes. Mol Biol Evol. 2007; 43: 986–998. doi: 10.1016/j.ympev.2006.06.009.
4. Chen WJ, Bonillo C, Lecointre G. 2003. Repeatability of clades as a criterion of reliability: a case study for molecular phylogeny of Acanthomorpha (Teleostei) with larger number of taxa. Mol Phylogenet Evol. 2003; 26: 262–288.
5. Meyer A, Lydeard C. The evolution of copulatory organs, internal fertilization, placentae, and viviparity in killifishes (Cyprinodontiformes), as inferred from a DNA phylogeny of the tyrosine kinase gene X-src Proc R Soc Lond B Biol Sci; 1993*.* 254: 153–162. doi: 10.1098/rspb.1993.0140.
