# Supplementary material for: Multigene phylogeny supports diversification of four-eyed fishes and one-sided livebearers (Cyprinodontiformes: Anablepidae) related to major South American geological events
Source: PLoS One. 2018 Jun 18;13(6):e0199201. doi: 10.1371/journal.pone.0199201 (PMC6005514; doi:10.1371/journal.pone.0199201)
Supplement: S3 Table — (DOC) [file pone.0199201.s003.doc]

S3 Table. Best evolutive model for each partition found by PartitionFinder.

| Partition | Codon positions | Best evolutive model |
| --- | --- | --- |
| 1 | Enc1_pos1; Rag1_pos3; Sh3px3_pos1; Rho_pos3; X-src_pos3 | GTR |
| 2 | Glyt_pos2; Myh6_pos1; Myh6_pos2; Rag1_pos1; Rho_pos2; | GTR+I |
|  | Sh3px3_pos2; Sh3px3_pos3 |  |
| 3 | Enc1_pos2 | HKY+I |
| 4 | Enc1_pos3 | TRNF |
| 5 | Glyt_pos3 | TRNF+I |
| 6 | Glyt_pos1 | TIM |
| 7 | Myh6_pos3 | SYM |
| 8 | Rag1_pos2 | TVM+I |
| 9 | Rho_pos1 | SYM+I |
| 10 | X-src_pos1 | K80+I |
| 11 | X-src_pos2 | F81+I |
